# Development and origins of Zebrafish ocular vasculature

**DOI:** 10.1186/s12861-015-0066-9

**Published:** 2015-03-27

**Authors:** Rivka Kaufman, Omri Weiss, Meyrav Sebbagh, Revital Ravid, Liron Gibbs-Bar, Karina Yaniv, Adi Inbal

**Affiliations:** Department of Medical Neurobiology, Institute for Medical Research - Israel-Canada, The Hebrew University-Hadassah Medical School, Jerusalem, Israel; Department of Biological Regulation, Weizmann Institute of Science, Rehovot, Israel; Department of Medical Neurobiology, Hebrew University Medical School, Ein-Kerem, Jerusalem 9112002 Israel

**Keywords:** Zebrafish, Eye, Ocular vasculature, Hyaloid vessels

## Abstract

**Background:**

The developing eye receives blood supply from two vascular systems, the intraocular hyaloid system and the superficial choroidal vessels. In zebrafish, a highly stereotypic and simple set of vessels develops on the surface of the eye prior to development of choroidal vessels. The origins and formation of this so-called superficial system have not been described.

**Results:**

We have analyzed the development of superficial vessels by time-lapse imaging and identified their origins by photoconversion experiments in *kdrl:Kaede* transgenic embryos. We show that the entire superficial system is derived from a venous origin, and surprisingly, we find that the hyaloid system has, in addition to its previously described arterial origin, a venous origin for specific vessels. Despite arising solely from a vein, one of the vessels in the superficial system, the nasal radial vessel (NRV), appears to acquire an arterial identity while growing over the nasal aspect of the eye and this happens in a blood flow-independent manner.

**Conclusions:**

Our results provide a thorough analysis of the early development and origins of zebrafish ocular vessels and establish the superficial vasculature as a model for studying vascular patterning in the context of the developing eye.

**Electronic supplementary material:**

The online version of this article (doi:10.1186/s12861-015-0066-9) contains supplementary material, which is available to authorized users.

## Background

The formation of correctly patterned vasculature is necessary for normal organ development and function. Two main mechanisms underlie formation of vascular networks: vasculogenesis and angiogenesis. In vasculogenesis, angioblasts, the precursors of endothelial cells, coalesce at a specific region where they differentiate into endothelial cells and rearrange to form new blood vessels. In angiogenesis, new blood vessels arise from an existing vessel, either by splitting of the vessel (intussusceptive angiogenesis) or by sprouting of new vessels (sprouting angiogenesis) [1,2].

The developing eye receives blood supply from the hyaloid and choroidal vascular systems. The hyaloid system is intraocular, and forms a dense network of vessels that reaches and partially covers the posterior aspect of the lens [3-5]. Hyaloid vessels are transient and are replaced by retinal vasculature [3]. The more superficial part of the developing eye receives blood supply through the choroidal system, which forms a plexus of capillaries that are located outside the retinal pigmented epithelium (RPE) layer and encircles the optic cup [3]. Abnormal formation of ocular vasculature in the embryo as well as abnormal vascularization in the mature eye are implicated in several blinding diseases [6,7], underscoring the importance of understanding the development of these vessels.

**Figure 1:**
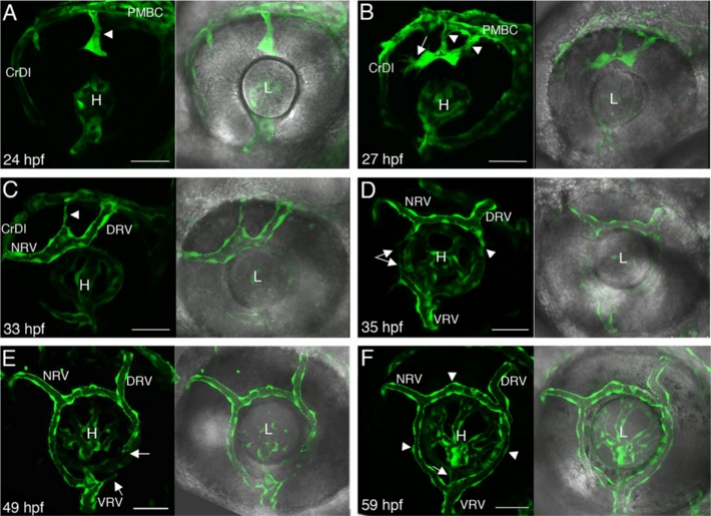
**Development of the superficial system. (A-F)** Projections of confocal z-stacks (left in each panel) showing ocular vessels in *Tg(kdrl:EGFP)* live embryos at different developmental time points, which are depicted in each panel. On the right side of each panel are the same conofocal images combined with bright field images showing position of vessels relative to eye tissues. **(A)** Arrowhead points at an initial sprout that will form the DRV. **(B)** Arrowheads point at two vessels, one or both will form the DRV. These vessels have connected and the tip cell (arrow) grows towards the CrDI. **(C)** The DRV and NRV have formed. One of the two initial vessels that sprouted from the PMBC is being pruned (arrowhead). **(D)** Sprouts arising from the VRV and NRV/SAV junction send long filopodial extensions towards each other (arrows). The posterior part of the SAV begins to grow ventrally (arrowhead). **(E)** The posterior SAV continues to grow ventrally, but there is only minor angiogenic activity from the VRV. Arrows point at tip cell of posterior SAV and small filopodial extension from the VRV. **(F)** The completed superficial system. Arrow points at the hyaloid vein and arrowheads at the SAV. All images are lateral views, anterior to the left, dorsal up. CrDI, cranial division of internal carotid artery; DRV, dorsal retinal vessel; H, hyaloid system; L, lens; NRV, nasal radial vessel; PMBC, Primordial midbrain channel; SAV, superficial annular vessel; VRV, ventral radial vessel. Scale bars are 50 μm.

Zebrafish embryos have a hyaloid system as well as a simple array of vessels on the surface of the eye, which develops by two days post-fertilization (dpf) and is referred to hereafter as the superficial system [4,8,9]. Development of the superficial system precedes the appearance of an elaborate network of choroidal vessels on the surface of the eye at 9 dpf [9]. It is currently unclear how superficial vessels are related to choroidal vessels. In the hyaloid system the first vessel to form is the hyaloid artery (HA), which, as in other vertebrates, enters the eye via the optic fissure before the latter closes [4,9]. The hyaloid artery branches into capillaries inside the eye and blood flowing in these vessels drains through the hyaloid vein, which exits the eye through the optic fissure and connects to the superficial system [5,9,10]. The branching pattern of hyaloid capillaries varies such that the hyaloid system appears slightly different in individual embryos. The superficial system comprises the nasal radial vessel (NRV) through which blood enters, and the dorsal radial vessel (DRV) and ventral radial vessel (VRV) through which blood drains from the system (naming according to [9]). These vessels are interconnected by a ring-shaped vessel, the superficial annular vessel (SAV) [9] (also see Figure 1F). Unlike the hyaloid system, superficial vasculature appears highly similar between embryos suggesting it develops in a highly stereotypic manner.

In contrast to the more extensive knowledge on hyaloid system development, little is known about the development of superficial ocular vessels and the earliest stages of their formation have not been described; hence the origins of these vessels, the dynamics of their formation and the signals that influence their development are unknown. Additionally, it is unknown whether there are molecular differences between vessels that are anatomically referred to as arteries or veins. Here we provide a thorough analysis of superficial system development from its earliest stages. We show that the entire system forms by angiogenesis from a venous origin and surprisingly, we also find that the hyaloid system has, in addition to its previously described arterial origin, a different source for its venous part. We show that endothelial cells of the NRV, which serves as the artery for the superficial system, turn on Notch pathway activity as they grow, suggesting they acquire arterial identity. Together, our data add new insights into the development of ocular vasculature and show that the zebrafish superficial vascular system can serve as a model for identifying mechanisms of vascular patterning.

## Results

### Development of superficial ocular vasculature in zebrafish

To obtain a detailed understanding of how superficial vasculature forms from its earliest stages, we studied development of these vessels over time by imaging transgenic *kdrl:EGFP* embryos, whose endothelial cells express EGFP [11]. At 20 hours post-fertilization (hpf) (22 somite-stage), the primordial midbrain channel (PMBC) [8] can be seen growing dorsoanteriorly along the posterior margin of the eye, whereas the cranial division of internal carotid artery (CrDI) [8] is growing in a dorsoposterior direction along the anterior margin of the eye. The PMBC meets and connects with the CrDI at approximately 21–22 hpf (see movie in Additional file 1). From 22–23 hpf, sprouting from the PMBC over the dorsal retina can be seen where the DRV will be located (Figure 1A showing a sprout at 24 hpf and movie in Additional file 1). Typically, 1–2 sprouts grow in a ventral-anterior direction. If two sprouts grow, they bridge and continue to grow anteriorly as a single vessel dorsal to the lens until reaching and connecting to the CrDI at approximately 31–33 hpf, thereby forming the NRV (Figure 1B,C; see movie in the Additional file 2) [12]. Often one of the two sprouts will be pruned, but occasionally two DRVs remain. At approximately the same time of NRV formation, ventral sprouting begins from the junction of the NRV and SAV, concomitant with dorsal sprouting from the VRV. These sprouts send long filopodial extensions and grow towards each other to form the anterior part of the SAV, which is completed at around 40 hpf (Figure 1D; see movie in Additional file 3). At approximately 35 hpf, ventral sprouting from the junction of the DRV and SAV, together with less pronounced dorsal sprouting from the VRV, begin to form the posterior part of the SAV (Figure 1E showing growth of posterior SAV at 49 hpf; see movie in Additional file 4), thus completing formation of the early superficial system at approximately 53–54 hpf (see Figure 1F for a completed system at 59 hpf).

Formation of the superficial system is independent of blood flow. We injected antisense morpholino oligonucleotides (MO) that block translation of *troponin T2a* gene (*tnnt2a*; *silent heart*), which is specifically expressed in the heart and is essential for heart contraction [13]. Despite lack of blood flow in the morphants, superficial vessels formed in a correct pattern (see below).

### A single venous origin for superficial vessels and a mixed origin for hyaloid vasculature

The documentation of superficial vessel development suggests that most of these vessels originate from the dorsal region of the PMBC, beginning with sprouting of the future DRV. However, the VRV is present before sprouts from the dorsal part of the system grow ventrally, suggesting it could have a different origin. Moreover, the superficial and hyaloid systems are connected via the hyaloid vein (HV), which raises the question of whether these systems have any shared origins. To clarify these issues we generated transgenic fish expressing the photoconvertible protein Kaede in endothelial cells under the regulation of *kdrl* promoter, and used this transgenic line to map the origins of all early ocular vessels.

**Figure 2:**
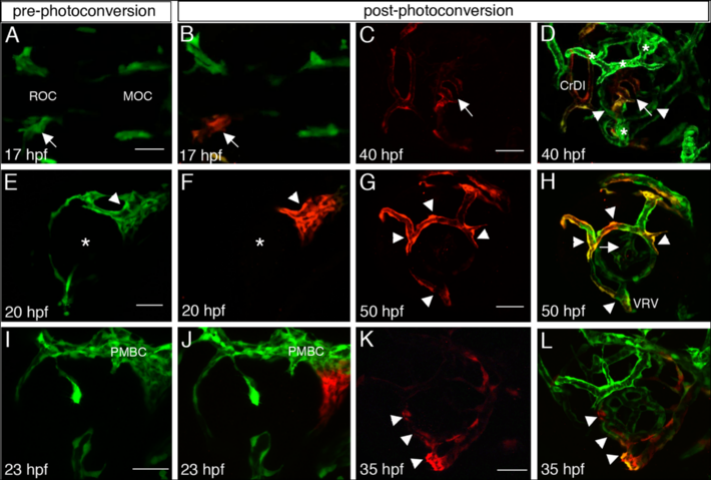
**Origins of superficial and hyaloid vessels. (A,E,I)** Pre-photoconversion and **(B-D, F-H, J-L)** post-photoconversion confocal z-stack projections of *kdrl:Kaede* embryos. Cells that were not photoconverteed are green whereas photoconverted cells are red. Age of embryos when imaged is depicted in each panel. **(A-D)** Photoconversion of left ROC. **(A,B)** Arrows point at the left ROC. **(C,D)** Single channel image showing only photoconverted endothelial cells **(C)**, and a merge of channels showing photoconverted and non-photoconverted cells **(D)**. Arrows and arrowheads point at central hyaloid vessels with photoconverted cells and more peripheral hyaloid vessels without photoconverted cells, respectively. **(E-H)** Photoconversion of PMBC. **(E,F)** Asterisks mark the center of the eye. **(G,H)** Single channel image showing only photoconverted endothelial cells **(G)**, and a merge of channels showing photoconverted and non-photoconverted cells **(H)**. Arrowheads point at superficial vessels and arrow in H points at hyaloid vessels. **(I-L)** Photoconversion of ventral PMBC. **(I,J)** Only the ventral region of the PMBC was photoconverted (red in **J**). **(K,L)** Single channel image showing only photoconverted endothelial cells **(K)**, and a merge of channels showing photoconverted and non-photoconverted cells **(L)**. Arrowheads point at the VRV and peripheral hyaloid vessels. **(A,B)** are dorsal views, all other panels are lateral views, anterior to the left. CrDI, cranial division of internal carotid artery; DRV, dorsal retinal vessel; MOC, midbrain organizing center; NRV, nasal radial vessel; PMBC, Primordial midbrain channel; ROC, rostral organizing center; VRV, ventral radial vessel. Scale bars are 50 μm.

All head vasculature develops from two clusters of angioblasts, which can be visualized from early segmentation stages: the rostral organizing center (ROC) and the midbrain organizing center (MOC) [14]. According to previous descriptions, posteriorly migrating ROC cells give rise to the CrDI, whereas anteriorly migrating cells give rise to the optic (ophthalmic) artery (OA) that grows into the eye to generate the hyaloid artery [8,14]. Consistent with the above mentioned previous studies [8,14], photoconversion of the entire ROC at 17 hpf (16-somite stage) (Figure 2A,B) and imaging at 40 hpf (Figure 2C,D) showed that the CrDI and central vessels of the hyaloid system were clearly labeled by photoconverted Kaede. Importantly, peripheral vessels of the hyaloid system, which are connected to the HV, as well as superficial vessels that developed by 40 hpf did not express photoconverted Kaede (Figure 2C,D) (n = 10/10). Photoconversion of endothelial progenitors migrating posteriorly or anteriorly from the ROC showed that these cells contributed to the CrDI or the central hyaloid vessels, respectively (data not shown). These results indicate that at least the central vessels of the hyaloid system originated from the ROC via the OA.

**Figure 3:**
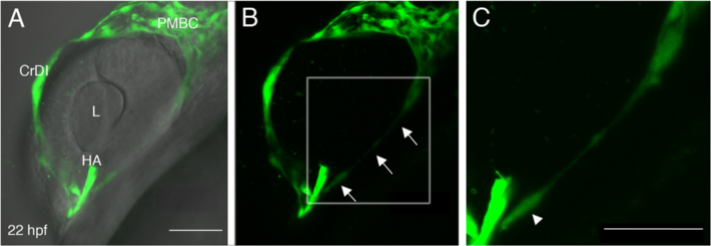
**Sprouting from the ventral PMBC. (A-C)** Projections of confocal z-stacks from a live *kdrl:EGFP* transgenic embryo at 22 hpf. In **A**, the same confocal image shown in **B** is combined with bright field image to demonstrate position of blood vessels relative to eye tissues. Arrows in **B** point at a long extension from the ventral PMBC towards the optic fissure, where the HA enters the eye. **(C)** A higher magnification of the region marked by white rectangle in **B** suggests the extension is actually a trail of cells. Arrowhead in **C** points at what appears to be an endothelial cell body. CrDI, cranial division of internal carotid artery; HA, hyaloid artery; L, lens; PMBC, Primordial midbrain channel. Anterior is to the left and dorsal up. Scale bars are 50 μm.

The PMBC is derived from the MOC [14] and, as we show above, gives rise to dorsal vessels of the superficial system. Indeed, when we photoconverted the entire PMBC at 20 hpf (22 somite-stage) and imaged embryos at 50 hpf, dorsal superficial vessels expressed photoconverted Kaede. Moreover, the VRV also contained photoconverted cells (Figure 2E-H) (n = 5/5), suggesting that the PMBC is the origin of all superficial vessels. In this experiment, none of the hyaloid vessels appeared to contain photoconverted cells. As the data suggested that the PMBC is also the source of the VRV, we re-examined growth of vessels from the PMBC. We noticed that during a short time window between 20–23 hpf, long extensions are sent from the ventral region of the PMBC towards the ventral optic fissure and growing hyaloid artery (Figure 3A,B). High magnification imaging suggests that these extensions are formed by a trail of individual endothelial cells, which migrate from the PMBC (Figure 3C). Indeed, when we repeated photoconversion specifically of the ventral PMBC at 22–23 hpf and imaged embryos at 35 hpf, we detected photoconverted cells in the VRV (Figure 2I-L). Surprisingly, peripheral hyaloid vessels were also photoconverted (Figure 2K,L) (n = 8/8), indicating that these vessels also originated from the PMBC. This conclusion is supported by time-lapse imaging showing growth of peripheral hyaloid vessels from the hyaloid vein and their connection to central hyaloid vessels that extend from the hyaloid artery, thereby demonstrating that these two groups of vessels indeed have different origins (see movie in Additional file 5). Together, the data show that the entire superficial system is of venous origin, the PMBC, whereas the hyaloid system originates both from arterial (OA and HA) and venous (PMBC) origins, which give rise to central and peripheral components of the system, respectively. The different origins of hyaloid vessels, together with direction of blood flow into the eye via the HA, through central vessels and then draining to the HV via peripheral vessels, raise the idea that central and peripheral hyaloid vessels represent arterial and venous components of the hyaloid system, respectively.

### The NRV appears to acquire arterial identity while growing

**Figure 4:**
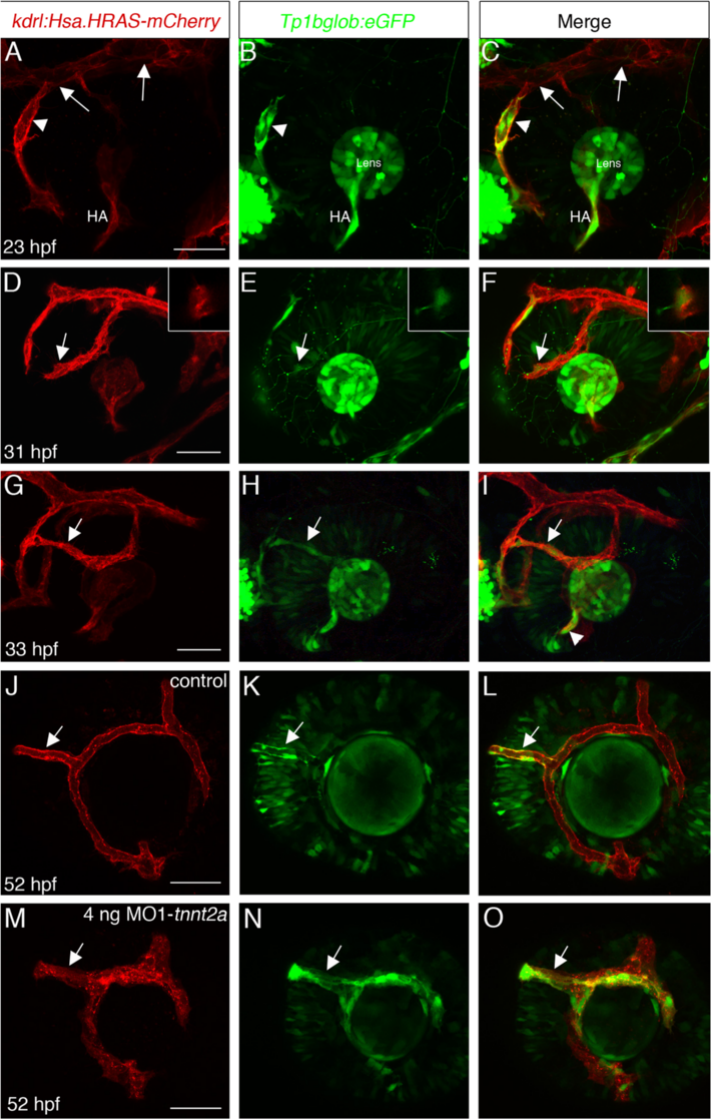
**Notch pathway activation in the NRV. (A-O)** Single channel or merged confocal z-stack projections of double transgenic embryos carrying *kdrl:Hsa.HRAS-mCherry* (red) and *Tp1bglob:eGFP* (green) transgenes. **(A-C)** At 23 hpf, the CrDI (arrowhead) expresses EGFP whereas the PMBC (arrows in **A** and **C**) does not. **(D-I)** At 31–33 hpf the NRV is close to connecting to the CrDI **(D-F)** or has already connected **(G-I)**. EGFP expression can be seen in one of the leading cells of the NRV (arrows in **D**-**F**). Insets are higher magnification of the region the arrows point at and are 4 μm single confocal sections. EGFP expression becomes clearer throughout the NRV once it is connected to the CrDI (arrows in **G**-**I**). **(J-O)** Control **(J-L)** and MO1-*tnnt2a* injected **(M-O)** embryos at approximately 52 hpf. EGFP expression in the NRV (arrows) is evident in injected embryos. HA, hyaloid artery. All images are lateral views, anterior to the left, dorsal up. CrDI, cranial division of internal carotid artery; DRV, dorsal retinal vessel; HA, hyaloid artery; NRV, nasal radial vessel; PMBC, Primordial midbrain channel; VRV, ventral radial vessel. Scale bars are 50 μm.

Given that the entire superficial system arises from a venous origin, the question arises whether the NRV, which according to direction of blood flow serves as the artery of this system [9], acquires at some point arterial identity. To address this question we first attempted to label embryos by in situ hybridization with various artery- or vein-specific markers. However, we could not detect any signals in superficial vessels, possibly due to their very small size and/or low levels of transcripts. We therefore asked if Notch signaling, which is known to be required for artery specification [15], was activated in the NRV. To observe Notch pathway activation we used the transgenic line *Tg(EPV.Tp1-Mmu.Hbb:EGFP)um14* (hereafter abbreviated as *Tp1bglob:eGFP*), which reports on Notch pathway activation by EGFP expression [16]. We used double transgenic embryos carrying *Tp1bglob:eGFP* and *kdrl:Hsa.HRAS-mCherry* transgenes to identify blood vessels showing Notch pathway activation. At 23 hpf the CrDI has connected to the PMBC, but superficial vasculature has not yet developed. Consistent with their artery and vein identities, the CrDI (an artery) expresses high levels of EGFP whereas no EGFP expression is detected in the PMBC (a vein) (Figure 4A-C). Moreover, the PMBC, but not the CrDI, expresses the vein-specific marker *dab2* [17,18], confirming that at this stage it already has a venous identity (Additional file 6). By approximately 31–33 hpf, the NRV connects to the CrDI. Imaging embryos shortly before this connection occurs revealed that EGFP expression is present in some of the leading cells of the growing NRV (Figure 4D-F) (n = 4/4). Immediately after it connects to the CrDI, EGFP expression can be clearly seen throughout the NRV (Figure 4G-I). These results show that the NRV, which arises from a vein, upregulates Notch pathway activity while growing, shortly before it connects to the CrDI. We hypothesize this activation of Notch reflects acquisition of arterial identity by the NRV. To further test this hypothesis we used a second transgenic line, *Tg(flt1_9a_cFos:GFP)wz2*, which utilizes a specific *flt1* enhancer previously described to drive strong expression of GFP in arteries and weak expression in veins [19]. Consistent with our hypothesis, at 52 hpf the NRV as well as few endothelial cells in the dorsal SAV expressed high GFP levels, whereas other superficial vessels expressed very low levels of GFP (n = 7/7) (Additional file 7). Moreover, central hyaloid vessels, which originate from an artery, also expressed high GFP levels whereas in peripheral hyaloid vessels, which originate from a vein, GFP expression was undetectable (Additional file 7). These results support the idea that the NRV acquires an arterial identity and that the hyaloid system has a central, arterial component and a peripheral, venous component.

It has been shown that in specific contexts, blood flow determines the identity of vessels [20]. Because Notch signaling is activated in the NRV before it connects to the CrDI, blood flow from the CrDI is not required for it to upregulate Notch pathway activity; however, it could be required for its maintenance. We therefore blocked blood flow by injecting *Tg(Tp1bglob:eGFP*; *kdrl:Hsa.HRAS-mCherry)* embryos with antisense morpholino oligonucleotides targeting *tnnt2* and examined them at 2 dpf. Despite lack of blood flow in injected embryos, the NRV expressed EGFP, indicating that maintenance of Notch activity was independent of blood flow (Figure 4M-O). Hence, the data suggest that local cues, rather than physiological changes, determine the apparent arterial identity of the NRV.

## Discussion and conclusions

**Figure 5:**
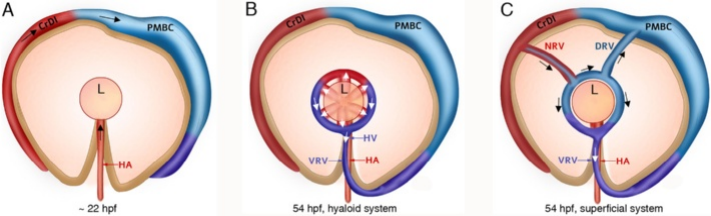
**Summary of ocular vessel origins. (A-C)** Schematic illustration of ocular vessel origin. The colors of vessels represent their origins: from the dorsal PMBC - light blue, ventral PMBC - purple and from the HA – red (excluding the CrDI). Blue and purple represent venous identity, red represents arterial identity. **(A)** Just before superficial vessels grow, the PMBC and CrDI have connected and the HA entered the eye through the optic fissure. **(B)** The hyaloid system. Vessels are located inside the eye, at a deeper level than vessels shown in **C**. **(C)** The completed superficial system. The NRV is red, indicating its arterial identity with a blue core representing its origin from the dorsal PMBC. Arrows in **B** and **C** show direction of blood flow. CrDI, cranial division of internal carotid artery; DRV, dorsal retinal vessel; HA, hyaloid artery; HV, hyaloid vein; L, lens; NRV, nasal radial vessel; PMBC, Primordial midbrain channel; SAV, superficial annular vessel; VRV, ventral radial vessel.

In this work we provide a detailed description of the development of zebrafish superficial ocular vasculature and identify the origins of hyaloid and superficial vessels (summarized schematically in Figure 5). Our results provide a basis for better understanding the molecular mechanisms that influence ocular vessel development.

The origins and development of hyaloid vessels have been previously described [4,5,8,9]. Consistent with these studies, our analyses show that central vessels of the hyaloid system originate from the OA. However, we also found, unexpectedly, that peripheral hyaloid vessels and the HV originate from the PMBC. Given that peripheral vessels empty into the HV and that the hyaloid system has two different origins for its central and peripheral vessels, we propose that central and peripheral parts of the system represent arterial and venous components, respectively. It will be interesting to find whether two distinct origins for hyaloid vessels are found in other vertebrates as well.

In contrast to the hyaloid system, superficial vessels have a single venous origin but the NRV acquires arterial identity, as judged by upregulation of Notch pathway activity and differential expression of GFP in *flt1_9a_cFos:GFP* transgenic embryos. This finding is consistent with the direction of blood flow in the NRV, suggesting it serves as the artery of the superficial system. Hence, unlike the hyaloid system where presumptive arteries and veins have arterial and venous origins, respectively, the superficial system presents a scenario where one of the vessels comprising it acquires an identity, which is different from its vessel of origin. This finding suggests there is plasticity in artery-vein specification. Indeed, such plasticity has been shown in chick embryos during a limited early time window in development [21,22]. Interestingly, change of identity from veins to arteries was also shown during formation of coronary arteries, that form from differentiated venous endothelial cells in the sinus venosus [23]. Together with the fact that the NRV upregulates Notch pathway activity as it grows and before connecting to the CrDI, these findings suggest that local cues in the environment promote this change of endothelial cell type. The most likely tissue to mediate the environmental cues is the developing eye on which superficial vessels grow and it will be challenging to identify the signals that direct both the patterning of superficial vessels and the molecular changes in the NRV.

Previous studies have identified genes and drugs that influence hyaloid system development without affecting development of other vascular systems, e.g. intersegmental vessels (ISVs) [4,9]. These results demonstrate heterogeneity in the molecular mechanisms that underlie hyaloid and ISV formation. It will be important to find whether similar heterogeneity exists between molecular mechanisms governing superficial vessel development and other vascular systems and whether superficial vessels and hyaloid vessels are similarly affected by specific molecular cues.

The simple, yet highly stereotypic pattern of the superficial vasculature, makes it an excellent model for studying vascular patterning. We hypothesize that the molecular landscape of the eye provides cues for directing the growing vessels. Eye tissues that are in close proximity to developing vessels are likely to influence their growth and include neural retina, retinal pigmented epithelium, lens and surface epithelium that will develop into cornea. Relevant molecular cues can be attractants or repellents, influencing vessels directly or acting as modifiers of an angiogenesis-promoting signal such as vascular endothelial growth factor (VEGF). Candidate molecules would be, for example, cell adhesion molecules and cell-cell signaling molecules. By manipulating expression of such molecules within eye tissues, it will be possible to gain new insights into the molecular mechanisms that shape vascular systems.

## Methods

### Fish lines

The following published lines were used in this work: AB and TL lines were used as wild type; *Tg(kdrl:EGFP)s843* [11]; *Tg(kdrl:Hsa.HRAS-mCherry)s896* [24]; *Tg(EPV.Tp1-Mmu.Hbb:EGFP)um14* [16]. New transgenic lines: For *Tg*(*kdrl:Kaede)huj9* line, a *kdrl:Kaede* construct was cloned in a Tol2-compatible vector (additional details will be provided upon request). The *Tg(flt1_9a_cFos:GFP)wz2* line was generated by cloning the previously identified zebrafish *flt1 9a* enhancer [19] into pGW_cFosGFP Gateway destination vector [25] using the Gateway methodology [26]. Transgenic fish were generated by co-injection of *kdrl:Kaede* or *flt1_9a_cFos:GFP* plasmid DNA and synthetic RNA encoding Tol2 transposase as described [27]. All research was conducted with approval of the Hebrew University Authority for Biological and Biomedical Models.

### In situ hybridization and morpholino injection

Whole-mount in situ hybridization (ISH) using riboprobes was performed according to standard protocols [28]. BMPurple (Roche) was used as blue substrate. *dab2* probe has been described [17]. MO1-*tnnt2a* [13] has been described and was injected at the 1–2 cell stage.

### Photoconversion and imaging

All photoconversion experiments and fluorescent imaging were performed using Zeiss LSM 700 confocal microscope. To block pigmentation when imaging embryos older than 24 hpf, embryos were raised in the presence of 0.003% *N*-Phenylthiourea (PTU) (Sigma, P7629) from approximately 22 hpf. For photoconversion and imaging, embryos were mounted in 0.5% low melting point agarose (Lonza, 50101) in 30% Danieau’s solution and 0.01% tricaine. Embryos were imaged before photoconversion using a 488 nm laser using the minimal necessary laser power. Photoconversion of Kaede was performed by scanning the selected region of interest (ROI) with a 405 nm laser. The scans were repeated until green fluorescence was eliminated and red fluorescence was confirmed and documented. Embryos were then released from the agarose and transferred to a petri dish with egg water containing PTU and covered with aluminum foil to protect from light. Embryos were kept at 28.5°C until they were imaged again.

For time-lapse analyses embryos were mounted as described above. Images were obtained every 10 minutes using the lowest laser power that provided a clear signal. In some cases development of filmed embryos was slightly slower than their non-filmed siblings, but vascular patterning and other aspects of development appeared completely normal. Images were exported as a movie using Zen software (Zeiss), with four frames per second. Each movie represents at least three similar movies from different embryos.

## Additional files

Additional file 1:
**Movie 1.** Earliest stages of ocular vessel development. The development of ocular vessels between approximately 18.5 hpf and 25 hpf. CrDI and PMBC grow towards each other and connect (first arrow) over the dorsal aspect of the eye. The hyaloid artery grows into the eye and the DRV sprouts (second arrow) from the PMBC. In this and following movies, time measurement depicted in top left corner is in hours and minutes. Additional file 2:
**Movie 2.** Formation of the NRV. Development of superficial vessels between approximately 28 hpf and 34 hpf. The DRV has already sprouted and can be seen growing towards and connecting to the CrDI, to form the NRV. Lumen formation in the DRV can be seen before connection to the CrDI is completed. Note pruning of one of the two early sprouts from the PMBC (arrow). Additional file 3:
**Movie 3.** Formation of the anterior SAV. Between approximately 33 hpf and 40 hpf, after NRV formation, dorsal and ventral sprouts from the NRV and VRV, respectively, grow towards each other (arrow) to form the anterior part of the SAV. Deeper frames showing the hyaloid system are not included in this movie due to overlap with some superficial vessels. In this movie too, pruning of one of the two early sprouts from the PMBC can be seen. Additional file 4:
**Movie 4.** Formation of the posterior SAV. Between approximately 41 hpf and 54 hpf, a single sprout grows ventrally to form the posterior SAV. A sprout growing from the VRV dorsally is also observed, although its contribution to the posterior SAV is typically smaller than the dorsal sprout. When both sprouts meet and connect, the initial superficial system is completed. Deeper frames showing the hyaloid system are not included in this movie due to overlap with some superficial vessels. Additional file 5:
**Movie 5.** Connecting peripheral and central hyaloid vessels. Between approximately 33 hpf and 40 hpf, a peripheral hyaloid vessel (blue arrow), which originates from the VRV, grows towards a central hyaloid vessel (red arrow) and they subsequently connect (white arrow). A and V at the end of the movie depict central (arterial) and peripheral (venous) components of the hyaloid system. Additional file 6:
***dab2*** **expression in the PMBC.** In situ hybridization for *dab2* expression at 22 hpf shows it is expressed in the PMBC (arrow) but not in the CrDI (arrowhead points to the location of the CrDI). Additional file 7:
***flt1_9a_cFos:GFP*** **transgene expression in ocular vessels.** (**A**-**C**) Single channel or merged confocal z-stack projections of 52 hpf double transgenic embryos carrying *kdrl:Hsa.HRAS-mCherry* (red) and *flt1_9a_cFos:GFP* (green) transgenes. High GFP expression indicating arterial identity is evident in the NRV (arrows) and central hyaloid vessels. Arrowheads in A and C point at peripheral hyaloid vessels that do not express GFP and are therefore undetectable in B. H, hyaloid system. Lateral views, anterior to the left. Scale bars are 50 μm.

## Abbreviations

Vascular nomenclature is based on [8, 9, 14]. CrDI: Cranial division of the internal carotid artery
DRV: Dorsal radial vessel
HA: Hyaloid artery
HV: Hyaloid vein
MOC: Midbrain organizing center
NRV: Nasal radial vessel
PMBC: Primordial midbrain channel
ROC: Rostral organizing center
SAV: Superficial annular vessel
VRV: Ventral radial vessel

## References

[1] Heinke J, Patterson C, Moser M (2012). Life is a pattern: vascular assembly within the embryo. Front Biosci (Elite Ed).

[2] Ellertsdottir E, Lenard A, Blum Y, Krudewig A, Herwig L, Affolter M (2010). Vascular morphogenesis in the zebrafish embryo. Dev Biol.

[3] Saint-Geniez M, D’Amore PA (2004). Development and pathology of the hyaloid, choroidal and retinal vasculature. Int J Dev Biol.

[4] Alvarez Y, Cederlund ML, Cottell DC, Bill BR, Ekker SC, Torres-Vazquez J (2007). Genetic determinants of hyaloid and retinal vasculature in zebrafish. BMC Dev Biol.

[5] Hartsock A, Lee C, Arnold V, Gross JM (2014). In vivo analysis of hyaloid vasculature morphogenesis in zebrafish: a role for the lens in maturation and maintenance of the hyaloid. Dev Biol.

[6] Gariano RF, Gardner TW (2005). Retinal angiogenesis in development and disease. Nature.

[7] Dorrell M, Uusitalo-Jarvinen H, Aguilar E, Friedlander M (2007). Ocular neovascularization: basic mechanisms and therapeutic advances. Surv Ophthalmol.

[8] Isogai S, Horiguchi M, Weinstein BM (2001). The vascular anatomy of the developing zebrafish: an atlas of embryonic and early larval development. Dev Biol.

[9] Kitambi SS, McCulloch KJ, Peterson RT, Malicki JJ (2009). Small molecule screen for compounds that affect vascular development in the zebrafish retina. Mech Dev.

[10] Weiss O, Kaufman R, Michaeli N, Inbal A (2012). Abnormal vasculature interferes with optic fissure closure in lmo2 mutant zebrafish embryos. Dev Biol.

[11] Jin SW, Beis D, Mitchell T, Chen JN, Stainier DY (2005). Cellular and molecular analyses of vascular tube and lumen formation in zebrafish. Development.

[12] Kochhan E, Lenard A, Ellertsdottir E, Herwig L, Affolter M, Belting HG (2013). Blood flow changes coincide with cellular rearrangements during blood vessel pruning in zebrafish embryos. PLoS One.

[13] Sehnert AJ, Huq A, Weinstein BM, Walker C, Fishman M, Stainier DY (2002). Cardiac troponin T is essential in sarcomere assembly and cardiac contractility. Nat Genet.

[14] Proulx K, Lu A, Sumanas S (2010). Cranial vasculature in zebrafish forms by angioblast cluster-derived angiogenesis. Dev Biol.

[15] Lawson ND, Scheer N, Pham VN, Kim CH, Chitnis AB, Campos-Ortega JA (2001). Notch signaling is required for arterial-venous differentiation during embryonic vascular development. Development.

[16] Parsons MJ, Pisharath H, Yusuff S, Moore JC, Siekmann AF, Lawson N (2009). Notch-responsive cells initiate the secondary transition in larval zebrafish pancreas. Mech Dev.

[17] Song HD, Sun XJ, Deng M, Zhang GW, Zhou Y, Wu XY (2004). Hematopoietic gene expression profile in zebrafish kidney marrow. Proc Natl Acad Sci U S A.

[18] Bradford Y, Conlin T, Dunn N, Fashena D, Frazer K, Howe DG (2011). ZFIN: enhancements and updates to the Zebrafish model organism database. Nucleic Acids Res.

[19] Bussmann J, Bos FL, Urasaki A, Kawakami K, Duckers HJ, Schulte-Merker S (2010). Arteries provide essential guidance cues for lymphatic endothelial cells in the zebrafish trunk. Development.

[20] le Noble F, Moyon D, Pardanaud L, Yuan L, Djonov V, Matthijsen R (2004). Flow regulates arterial-venous differentiation in the chick embryo yolk sac. Development.

[21] Moyon D, Pardanaud L, Yuan L, Breant C, Eichmann A (2001). Plasticity of endothelial cells during arterial-venous differentiation in the avian embryo. Development.

[22] Othman-Hassan K, Patel K, Papoutsi M, Rodriguez-Niedenfuhr M, Christ B, Wilting J (2001). Arterial identity of endothelial cells is controlled by local cues. Dev Biol.

[23] Red-Horse K, Ueno H, Weissman IL, Krasnow MA (2010). Coronary arteries form by developmental reprogramming of venous cells. Nature.

[24] Chi NC, Shaw RM, De Val S, Kang G, Jan LY, Black BL (2008). Foxn4 directly regulates tbx2b expression and atrioventricular canal formation. Genes Dev.

[25] Fisher S, Grice EA, Vinton RM, Bessling SL, Urasaki A, Kawakami K (2006). Evaluating the biological relevance of putative enhancers using Tol2 transposon-mediated transgenesis in zebrafish. Nat Protoc.

[26] Villefranc JA, Amigo J, Lawson ND (2007). Gateway compatible vectors for analysis of gene function in the zebrafish. Dev Dyn.

[27] Kawakami K, Takeda H, Kawakami N, Kobayashi M, Matsuda N, Mishina M (2004). A transposon-mediated gene trap approach identifies developmentally regulated genes in zebrafish. Dev Cell.

[28] Thisse C, Thisse B, Schilling TF, Postlethwait JH (1993). Structure of the zebrafish snail1 gene and its expression in wild-type, spadetail and no tail mutant embryos. Development.

