# Supplementary figures and images for: Development and origins of Zebrafish ocular vasculature
Source: BMC Dev Biol. 2015 Mar 27;15:18. doi: 10.1186/s12861-015-0066-9 (PMC4406013; doi:10.1186/s12861-015-0066-9)

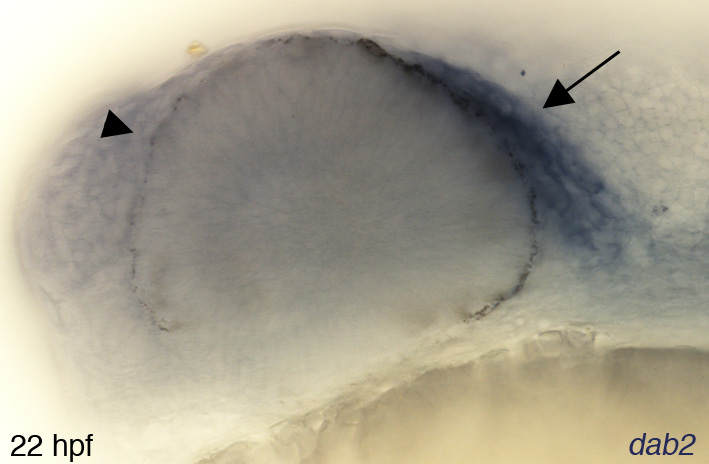

Supplement: Additional file 6: — dab2 expression in the PMBC. In situ hybridization for dab2 expression at 22 hpf shows it is expressed in the PMBC (arrow) but not in the CrDI (arrowhead points to the location of the CrDI). [file 12861_2015_66_MOESM6_ESM.jpeg]

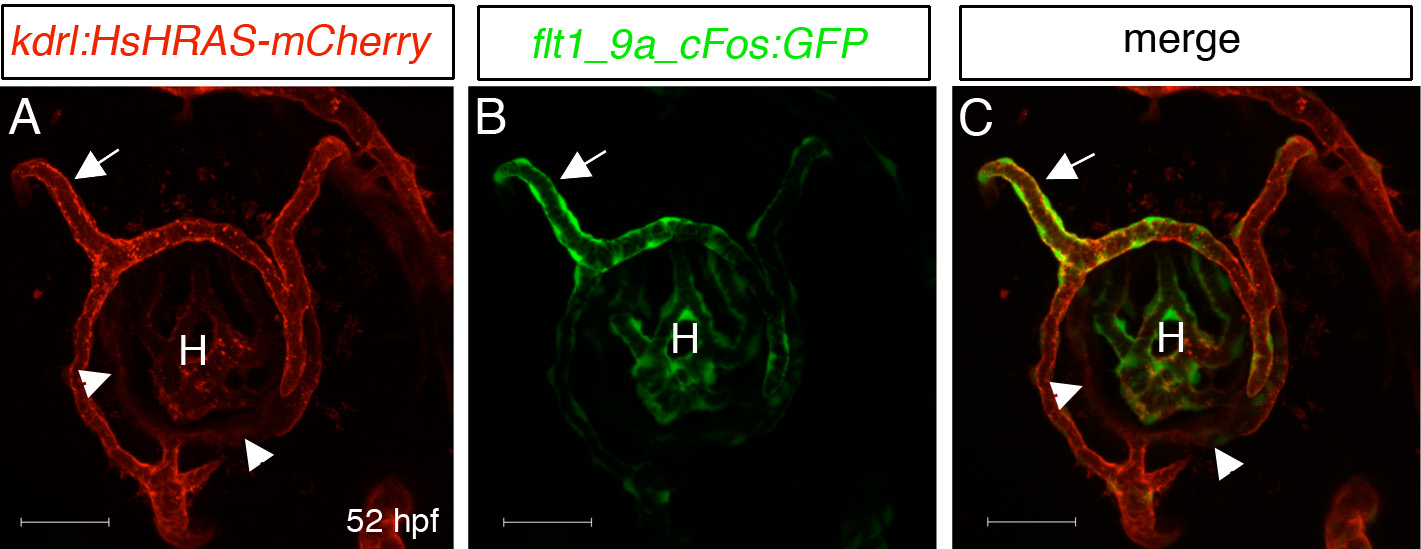

Supplement: Additional file 7: — flt1_9a_cFos:GFP transgene expression in ocular vessels. (A-C) Single channel or merged confocal z-stack projections of 52 hpf double transgenic embryos carrying kdrl:Hsa.HRAS-mCherry (red) and flt1_9a_cFos:GFP (green) transgenes. High GFP expression indicating arterial identity is evident in the NRV (arrows) and central hyaloid vessels. Arrowheads in A and C point at peripheral hyaloid vessels that do not express GFP and are therefore undetectable in B. H, hyaloid system. Lateral views, anterior to the left. Scale bars are 50 μm. [file 12861_2015_66_MOESM7_ESM.jpeg]
